# 4.1N-Mediated Interactions and Functions in Nerve System and Cancer

**DOI:** 10.3389/fmolb.2021.711302

**Published:** 2021-09-13

**Authors:** Qin Yang, Jing Liu, Zi Wang

**Affiliations:** ^1^ Molecular Biology Research Center & Center for Medical Genetics, School of Life Sciences, Central South University, Changsha, China; ^2^ School of Medical Laboratory, Shao Yang University, Shaoyang, China

**Keywords:** 4.1N, FERM, SAB, CTD, nerve system, cancer 2

## Abstract

Scaffolding protein 4.1N is a neuron-enriched 4.1 homologue. 4.1N contains three conserved domains, including the N-terminal 4.1-ezrin-radixin-moesin (FERM) domain, internal spectrin–actin–binding (SAB) domain, and C-terminal domain (CTD). Interspersed between the three domains are nonconserved domains, including U1, U2, and U3. The role of 4.1N was first reported in the nerve system. Then, extensive studies reported the role of 4.1N in cancers and other diseases. 4.1N performs numerous vital functions in signaling transduction by interacting, locating, supporting, and coordinating different partners and is involved in the molecular pathogenesis of various diseases. In this review, recent studies on the interactions between 4.1N and its contactors (including the α7AChr, IP3R1, GluR1/4, GluK1/2/3, mGluR8, KCC2, D2/3Rs, CASK, NuMA, PIKE, IP6K2, CAM 1/3, βII spectrin, flotillin-1, pp1, and 14-3-3) and the 4.1N-related biological functions in the nerve system and cancers are specifically and comprehensively discussed. This review provides critical detailed mechanistic insights into the role of 4.1N in disease relationships.

## Background

Neuron-enriched protein 4.1N and three other homologues (4.1R, 4.1B, and 4.1G) belong to the protein 4.1 family. The protein 4.1N is expressed in most animal cell types and tissues with different cell abundances. 4.1N is encoded by the gene *EPB41L1*, which undergoes tissue-specific alternative splicing. The 135-kDa isoform and the 100-kDa isoform of 4.1N are predominantly expressed in the brain and peripheral tissues, respectively (Walensky et al., 1999). 4.1N is the only non-erythroid protein (Baines et al., 2009; Fagerberg et al., 2014). 4.1R is predominantly characterized in erythrocytes and also expressed in numerous non-erythroid cells (Steck, 1974). 4.1B is highly expressed in the brain, kidney, testis, and intestine (Fagerberg et al., 2014). qPCR analysis reveals a predominate expression of 4.1G in the brain, spinal cord, and testis (Yang et al., 2011). The regulation and function for 4.1R, 4.1B, and 4.1G have been extensively characterized; much less is known about the regulation and function of 4.1N.

**FIGURE 1:**
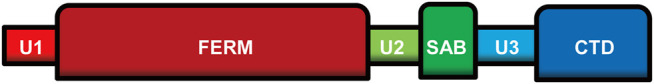
4.1N domain arrangement in vertebrates. FERM, N-terminal 4.1-ezrin-radixin-moesin domain; SAB, spectrin–actin–binding domain; CTD, C-terminal domain; U1/2/3, unique domain 1/2/3.

It has been proposed that the common activity of 4.1 proteins depends on their interaction with multiple membrane proteins and their assembly into macromolecular complexes (Baines et al., 2014). The spectrum of 4.1N interacting partners partially overlaps with other 4.1 homologues, because the structures of all 4.1 family members in vertebrates share a common domain pattern. The protein 4.1N has three conserved domains, including the N-terminal 4.1-ezrin-radixin-moesin (FERM) domain, internal spectrin–actin–binding (SAB) domain, and C-terminal domain (CTD), separated by three nonconserved unique domains (U1, U2, and U3) (Figure 1). The FERM and CTD domains of the protein 4.1N have essential roles in modifying synaptic plasticity, synaptic transmission, the spectrin/actin cytoskeleton profile, cell proliferation, cell adhesion, and signaling transduction. Unlike the SAB domain of 4.1B, 4.1G, and 4.1R, which evolves to act as a cytoskeletal linkage, 4.1N cannot form a ternary complex with spectrin and actin through the SAB domain (Gimm et al., 2002). This results from the fact that essential terminal hydroxyl-containing residue, serine, or threonine, is replaced by a proline residue, thereby significantly changing the ES/T motif of the 4.1N SAB domain.

Many molecules, including α7 acetylcholine receptor (α7AChr) (Kanno et al., 2013), inositol 1,4,5-trisphosphate receptor type 1 (IP3R1) (Zhang et al., 2003; Fukatsu et al., 2004; Fukatsu et al., 2006; Fiedler and Nathanson, 2011), GluR1/4 ^13,14^, GluK1/2/3 ^15,16^, metabotropic glutamate receptor 8 (mGluR8) (Rose et al., 2008), K-Cl co-transporter 2 (KCC2) (Li et al., 2007; Horn et al., 2010), D2 and D3 dopamine receptors (D2/3Rs) (Binda et al., 2002; Kabbani and Levenson, 2006), Ca2^+^-calmodulin serine kinase (CASK) (Cohen et al., 1998; Biederer and Sudhof, 2001; Mburu et al., 2006), nuclear mitotic apparatus (NuMA) (Ye et al., 1999; Ilies et al., 2012), phosphoinositide 3 kinase enhancer (PIKE) (Ye et al., 2000), inositol hexakisphosphate kinase 2 (IP6K2) (Nagpal et al., 2018), cell adhesion molecule (CAM) 1/3 ^29,30^, βII spectrin (Wang et al., 2018), flotillin-1 ^32^, pp1 (Wang et al., 2016), and 14-3-3 ^34-36^, have been identified as 4.1N binding partners. Although 4.1N performs different functions depending on specific tissue localization, 4.1N predominantly functions as a scaffolding protein in signaling transduction by locating, supporting, and coordinating multiple partners. Furthermore, 4.1N abnormality induces mislocalization and/or dysfunction of its partner and leads to the emerging role of 4.1N as an essential player in nervous cell function and tumor suppression. 4.1N-mediated protein–protein interactions and biological functions in the nerve system and cancers are specifically and comprehensively described in this review, contributing to understanding the role of 4.1N in disease relationships and also giving a brief understanding of the other 4.1 homologues.

## 4.1N in the Nerve System

### A-Amino-3-Hydroxy-5-Methyl-4-Isoxazole Propionate Receptors

Glutamate receptors are the major mediators of excitatory neurotransmitters in the mammalian central nervous system (Hollmann and Heinemann, 1994). Glutamate receptors are classified into 4 subtypes, including a-amino-3-hydroxy-5-methyl-4-isoxazole propionate receptors (AMPARs), kainate receptors (KARs), N-methyl-D-aspartate receptors, and G protein-coupled glutamate receptors (Shen et al., 2000).

4.1 homologues 4.1N and 4.1G are suggested to have a joint role in binding and regulating synaptic trafficking of the AMPAR subunits GluR1 and GluR4. 4.1N colocalizes with AMPARs at excitatory synapses. It is speculated that the FERM domain of 4.1N (Lin et al., 2009) binds with two separate regions in the C-terminal of AMPAR subunits GluR1 and GluR4: the 14-residue proximal segment and a more distal unidentified segment, and this region is critical to localize and stabilize GluR1/4-containing AMPARs in neural cells (Coleman et al., 2003). Interestingly, 4.1N does not bind with the C-terminal domains of GluR2 and GluR3, though the 14-residue segments of GluR1 and GluR4 have less sequence identity than GluR4 with either GluR2 or GluR3. Besides the amino acid sequence, the structural determinants of the segments are likely to contribute to the 4.1N interactions. Several regions of high sequence identity beyond the 14-residue segment in the C-terminal are shared by GluR1 and GluR4 but not found in GluR2 and GluR3. The 4.1G FERM domain interacts with the C-terminal of GluR1 and GluR4 (Shen et al., 2000; Wozny et al., 2009). The CTD overexpression of 4.1N and 4.1G or disruption of the F-action network leads to reduced plasma membrane GluR1 both in heterologous cells and cultured neurons, suggesting that the proteins 4.1N and 4.1G act as a link between AMPARs and the actin cytoskeleton, through which 4.1N anchors AMPARs to the actin cytoskeleton (Coleman et al., 2003), stabilizes AMPAR expression on the excitatory synapse surface (Coleman et al., 2003), and contributes to synaptic plasticity (Lin et al., 2009). However, *in vitro* data of 4.1N–GluR1/GluR4 interaction are not consistent with the *in vivo* situation. *In vivo*, 4.1G and 4.1N do not play crucial roles in glutamatergic synaptic transmission and maintaining long-term plastic changes in synaptic efficacy. The functional decline of glutamatergic synapses is not observed in 4.1G/N double-mutant mice. Some AMPAR scaffold proteins substitute the 4.1G/N loss, which is the possible reason for the lack of phenotypic changes in 4.1G/N double-mutant mice (Wozny et al., 2009; Zhang et al., 2014). Activation of GluR1-containing nucleus accumbens AMPARs in subregions of the brain nucleus accumbens enhanced the reinstatement of cocaine seeking. Although 4.1N contributes to GluR1 trafficking and stabilization in synapses, preventing endogenous 4.1N binding with GluR1 subunits in the accumbal subregion of the medial accumbens shell does not affect cocaine seeking (White et al., 2016).

The 4.1N–AMPAR bindings may not simply explain the AMPARs’ recruitment activity to synapses. 4.1N interacts with various proteins that are important for maintaining a balance in the dynamics of AMPARs trafficking through 4.1N (Chevy et al., 2015; Kesaf et al., 2020). The GluK2 KAR subunits, KCC2 and CAM 1, are 4.1N-interacting proteins. KCC2 also directly binds to GluK2 (Kesaf et al., 2020). In KCC2-deficient neurons, impaired trafficking activity of AMPARs to synapses is documented (Gauvain et al., 2011; Fiumelli et al., 2013; Chevy et al., 2015), which is the downstream role of KCC2 in the spine maturation (Kesaf et al., 2020). On the other hand, GluK2 deficiency leads to a significant change in subcellular distribution of KCC2 and reduction of GluR2 (Kesaf et al., 2020). 4.1N expression is significantly downregulated in GluK2−/− mice (Copits and Swanson, 2013) and cultured hippocampal neurons transduced with GluK2 shRNA (Kesaf et al., 2020). The relationship between 4.1N, KCC2, and Gluk2 in modulating AMPAR recruitment needs to be further explored. Synapse-associated protein-97 (SAP97) also regulates the synaptic localization of AMPARs. 4.1N potentially binds SAP97 isoforms containing the I3 domain. The endogenous 4.1N colocalizes with the SAP97 in hippocampal neurons, especially at synapses (Rumbaugh et al., 2003). Moreover, the expressions of 4.1N, SAP97, and GluR1 are regulated in the same way during rat cerebellar development (Douyard et al., 2007). The three proteins are observed mostly on neurons and are undetectable on glia in early postnatal days but shift from neurons to Bergmann glia after a few weeks (Douyard et al., 2007). 4.1N is associated with AMPARs (Lin et al., 2009), while synaptic protein SAP97 binds to AMPARs (Rumbaugh et al., 2003) and potentially interacts with 4.1N (Lue et al., 1994); thereby, a complex of 4.1N–AMPAR–SAP97 for AMPARs trafficking at synapses may coexist (Rumbaugh et al., 2003). Finally, CAM1 is an important molecule during early synaptogenesis, and 4.1N is a specific CAM1 effector for AMPAR recruitment during synapse formation (Hoy et al., 2009).

### Kainate Receptors

GluK1, GluK2, and GluK3 receptors are composed of low-affinity subfamily KARs (Valbuena and Lerma, 2019). 4.1N binds to a membrane-proximal domain of the C-terminal of GluK1/2/3 KAR subunits and regulates receptor trafficking, synaptic targeting, and endocytosis (Copits and Swanson, 2013). Although 4.1N is abundantly expressed in dendritic spines, inhibiting 4.1N interaction with the KARs leads to KAR lost on the cell surface along the dendritic shaft at a subset of spines only (Copits and Swanson, 2013). Additional 4.1 proteins, 4.1B and 4.1G, are expressed in the brain, and it remains unknown if these 4.1 homologues have specific functions in targeting KARs.

The palmitoylation and phosphorylation are dynamic and contribute to activity-dependent regulation of protein subcellular distribution and interactions in cells (Noritake et al., 2009). Regulation of the interaction with 4.1N by palmitoylation and phosphorylation is considered as a central mechanism for correlated but opposing alterations in AMPAR and KAR signaling (Hirbec et al., 2003; Noritake et al., 2009; Copits and Swanson, 2013). 4.1N-KARs and 4.1N-AMPARs act in an opposing manner, maintaining a pool of surface receptors along the dendritic shaft and the extrasynaptic membrane (Copits and Swanson, 2013). Palmitoylation within the proximal site (858 and 871 residues) of GluK2-containing KARs promotes the 4.1N–GluK2 binding and their expressions on the neuronal surface but compromises the receptor endocytosis. On the contrary, protein kinase C (PKC) phosphorylation of the proximal (S846 residue) and distal (S868 residue) sites of GluK2a-containing KARs synergistically and negatively regulates the neuronal 4.1N–GluK2a interactions in acute brain slices, which can be reversed after PKC inhibition. Whereas proximal phosphorylation of the GluR1 C terminus (S831, S816, and S818 residues) (Hayashi et al., 2000; Lin et al., 2009) is permissive for 4.1N–GluR1 binding, palmitoylation within the C domain (811 residue) (Hayashi et al., 2005; Lin et al., 2009) instead antagonizes 4.1 N binding to GluR1 and consequently stabilizes on the plasma membrane. The surface expression and extrasynaptic insertion of the AMPA receptor GluR1 and GluR4 subunits are regulated by 4.1N, which is mediated by associations of 4.1N and receptor membrane-proximal domains (Shen et al., 2000; Coleman et al., 2003; Lin et al., 2009) and has an analogous primary sequence with KAR subunits (Copits and Swanson, 2013). Indirect interaction with 4.1N as part of a larger macromolecular signaling complex, including 4.1N, GluK2-containing KARs, and GluR1/GluR4 AMPARs, is possible (Copits and Swanson, 2013).

Intriguingly, 4.1N may also be involved in PKC phosphorylation and SUMOylation of GluK2 KARs to regulate the surface expression and function of GluK2 KARs. PKC phosphorylation of GluK2a KARs (S868 and S846 residues) causes 4.1N–GluK2a disassociation and receptor endocytosis; thus, fewer GluK2a KARs are expressed on the neuronal surface (Copits and Swanson, 2013). PKC-dependent phosphorylation of GluK2 KARs (Ser868, but not Ser846 residue) activates SUMOylation (K886 residue) and subsequently promotes the removal of GluK2 KARs from the plasma membrane at the mossy fiber–CA3 synapses in the hippocampus during the long-term depression of KAR synaptic transmission (Konopacki et al., 2011; Chamberlain et al., 2012). Conversely, PKC-dependent phosphorylation (S868, but not S846 residue), without SUMOylation (K886 residue), leads to a more surface localization of GluK2 KARs (Chamberlain et al., 2012). It remains to be further clarified whether and how 4.1N and these post-translational modifications work in concert for fine-tuning of receptor expression and function.

The FERM domain of 4.1R contains phosphorylation sites and is regulated by PKC, protein kinase A, caseine, and tyrosine kinase (Manno et al., 2005; Gauthier et al., 2011). 4.1N also contains the conserved phosphorylation sites (Pearson and Kemp, 1991; Calinisan et al., 2006; Olsen et al., 2006; Zhang et al., 2014). In comparison with the 4.1N contractors, whether the protein 4.1N itself is phosphorylated in the phosphorylation events above is still unknown. However, the Trk receptor that directly phosphorylates the tyrosine residue of 4.1N and mediates 4.1N insertion into the nucleus under nerve growth factor (NGF) stimulation has been referred to in PC12 cells (Ye et al., 1999). A study focused on protein Ser/Thr-phosphorylation modifications in mice brain hemispheres underlying hereditary Parkinson’s disease has documented 2.2-fold increases of pS541, pS544, and pS546 in 4.1N protein (Auburger et al., 2019).

### D2 and D3 Dopamine Receptors

The D2/3Rs belong to the G protein-coupled glutamate receptor family. All 4.1 family members can bind to the IC3 domain within the N-terminal portion of the D2 (211–241 residues) and D3 (211–227 residues) dopamine receptors. 4.1N links the D2/D3Rs to the cytoskeleton and the 4.1N–D2/D3R interactions are necessary for distribution or stability of the D2/D3Rs on the cell membrane (Binda et al., 2002).

4.1N is suggested to be a novel target in antipsychotic drug development. It is conceivable that antipsychotic-induced 4.1N alteration, in turn, promotes modification of dopaminergic transmission and cytoskeleton profiles in neurons. The protein 4.1N is significantly increased in the mouse cortex after injecting either of the prototypical antipsychotic drugs haloperidol or clozapine (Kabbani and Levenson, 2006).

### Metabotropic Glutamate Receptor 8

mGluR 1–8 are subtypes of the G protein-coupled glutamate receptor and are distributed throughout the central nervous system. 4.1N, 4.1B, 4.1G, and 4.1R are highly expressed in synaptic layers of the retina, where they colocalize with mGluR8. In agreement with the specific colocalization, the intracellular mGluR8 C-terminus can bind all 4.1 homologues with different affinities (Rose et al., 2008). 4.1 homologues play a role in retinal function and development. Deficiency of 4.1 proteins leads to mislocalization of synapse components in the mouse retina (Rose et al., 2008).

### α7 Acetylcholine Receptor

4.1N is a binding partner of the α7AChr, which is mediated with the 4.1N CTD. The knock-down of 4.1N suppresses the plasma membrane location of α7AChr on PC-12 cells (Kanno et al., 2013). DCP-LA is a selective cytosolic PKC activator (Kanno et al., 2015). Particularly, DCP-LA increases the binding of 4.1N to α7AChr and α7AChr distribution to the plasma membrane in a 4.1N-dependent manner under PKC control, and this effect can be halted by 4.1N knock-down, however, irrespective of serine or threonine phosphorylation of 4.1N (Kanno et al., 2012; Kanno et al., 2013).

### Inositol 1,4,5-Trisphosphate Receptor Type 1

IP3R1 is the principal intracellular channel that mediates Ca2+ release from the endoplasmic reticulum (Kerkhofs et al., 2018). 4.1N and its binding partner IP3R1 are identified in rat brain synaptosomes. Both the C-terminal 14 amino acids of the cytoplasmic tail (CTT14aa) and the cytoplasmic tail middle 1 sequence of the IP3R1 have a binding affinity for 4.1N in peptide fragment forms. Nevertheless, only the CTT14aa in the IP3R1 full-length tetramer form is capable of binding with 4.1N (Fukatsu et al., 2006). 4.1N acts as a linkage between IP3R1 and actin filaments, spatiotemporally regulates lateral diffusion of the intracellular Ca2+ release channel in neuronal dendrites (Fukatsu et al., 2004), and mediates neurite formation through intracellular Ca2+ waves (Fiedler and Nathanson, 2011). The 4.1N–IP3R1 association is found in sub-confluent and confluent Madin–Darby canine kidney (MDCK) cells. When sub-confluent MDCK cells grow to confluent MDCK cells, the CTD of 4.1N is necessary and sufficient for the 4.1N–IP3R1 binding, and the FERM domain of 4.1N is responsible for translocating IP3R1 from the cell plasma to the basolateral membrane (Zhang et al., 2003). Notably, unlike in neurons and MDCK cells, 4.1N does not target IP3R1 in hepatocytes (Sehgal et al., 2005).

### K-Cl Co-Transporter 2

4.1N and KCC2 are highly expressed during the early development of neurons (Walensky et al., 1999; Gulyás et al., 2001; Ludwig et al., 2003). 4.1N in conjunction with KCC2 is significantly correlated with the maturation of excitatory synapses (Rivera et al., 1999; Li et al., 2007). 4.1N is a link between KCC2 and the dendritic spine cytoskeleton, and the FERM domain of 4.1N and the CTD of KCC2 are critical for mediating the direct interaction between 4.1N and KCC2 (Li et al., 2007). The interaction of 4.1N and KCC2 plays an essential role during the development of neurons. The perturbing binding of 4.1N to KCC2 results in an abnormal morphology of dendritic protrusion *in vitro* that is similar to the previous KCC2-deficient mice neurons (Li et al., 2007). An altered distribution pattern of 4.1N and actin is also obtained in the neural stem cell line C17.2 with ectopic expression of KCC2 (Horn et al., 2010). Similarly, when employing a neural-specific overexpression of KCC2 in neuronal progenitors of transgenic mouse embryos, aberrant cytoplasmic distributions of 4.1N and actin are found in the neural tube (Li et al., 2007; Horn et al., 2010). In addition, GluK2 loss induces a significant reduction of 4.1N and change of subcellular distribution of KCC2, as well as a smaller somatodendritic gradient (Kesaf et al., 2020). However, a mutated variant of KCC2 that cannot bind 4.1N does not affect cell morphology and the embryo phenotype (Horn et al., 2010).

### Ca2+-Calmodulin Serine Kinase

CASK is a membrane-associated guanylate kinase protein. The binding site of CASK for 4.1N^22^ and 4.1R^23^ locates at a HOOK region, between the Src homology 3 and guanylate kinase domains within the C-terminus. This binding facilitates F-actin nucleation at intercellular junctions formed by neurexins in neurons and acts in a salt-resistant and temperature-dependent manner (Biederer and Sudhof, 2001).

Both the 4.1N and CASK are confined to the stereocilia and expressed with an identical pattern on the hair cell surface in the inner ear, where it is vital for hearing. Although whirlin and shaker 2 are also located at the stereocilia bundle structure and critical for stereocilia development, neither 4.1N nor protein CASK expression is affected by the absence of whirler or shaker 2 (Mburu et al., 2006). 4.1R is also detected in the stereocilia bundle and partially colocalizes with whirlin at the stereocilia tip. 4.1R interacts with membrane palmitoylated protein (MPP) 1 in erythrocytes and stereocilia structures with an identical pattern (Alloisio et al., 1993; Mburu et al., 2006). Mutations in the *whirlin* and the *shaker2* genes lead to early ablation of 4.1R and MPP 1 labeling of stereocilia (Mburu et al., 2006).

### Inositol Hexakisphosphate Kinase 2

IP6K2 shows a high binding affinity to 4.1N and 4.1B and much less to 4.1R and 4.1G in mouse brain lysates, suggesting a relatively selective binding with 4.1N. The selective nature of the IP6K2–4.1N association is not observed in the bindings of 4.1N to IP6K1 or IP6K3 ^28^. 4.1N directly binds to the 202–261 residents of IP6K2. The interaction between 4.1N and IP6K2 in granule cells of the cerebellum regulates the Purkinje cell morphology and cerebellar synapses and is considered as a major determinant of cerebellar disposition and psychomotor behavior. 4.1N deficiency of cerebellar neurons impairs cell viability. The weakened interaction of 4.1N and IP6K2 in IP6K2-knockout mice elicits a notable impairment of motor coordination, a major cerebellar function (Nagpal et al., 2018). Increased mortality and movement defects are also observed in 4.1N−/− mice (Wang et al., 2020b). Nuclear translocation of 4.1N acts in an IP6K2-dependent manner in cerebellar granule cells, which is critical to performing main functions. Both 4.1N and IP6K2 are selectively abundant in cerebellar granule cells. Purkinje cells are the principal targets of granule cells (Nagpal et al., 2018). However, how 4.1N affects Purkinje cell morphology and function is still elusive.

IP6K2 is a predominantly p53-dependent proapoptotic enzyme. *IP6K2* gene deletion leads to a predisposition to tumor formation (Morrison et al., 2001; Rao et al., 2015). 4.1N is lost and acts as a suppressor in many tumors. It remains to be established how the 4.1N–IP6K2 interaction functions in tumors.

### Cell Adhesion Molecules

The CAM family plays critical roles in synaptic pruning, plasticity, stabilization (Duncan et al., 2021), tumor-associated pathways (Duraivelan and Samanta, 2021), etc. Loss of 4.1B in the axon is associated with reduced levels of CAM1 and CAM3 (Einheber et al., 2013). 4.1B and 4.1N are identified as specific CAM1 effector molecules for the recruitment of N-methyl-D-aspartate receptors and AMPARs to adhesion sites of synapses during synapse formation, respectively (Funaki et al., 2021). Protein 4.1 family members exhibit substantial homologies in the FERM (72–81% identity), SAB (53–66% identity), and CT (72–74% identity) domains (Parra et al., 2004). Why do homologues 4.1N and 4.1B show completely opposite specificities for glutamate receptor subtypes? The FERM domains of 4.1N and 4.1B share a 73% amino acid sequence identity. The 4.1B FERM domain is associated with CAM1 in kidney renal clear cell carcinoma (KIRC) (Yamada et al., 2006; Nagata et al., 2012), non–small-cell lung cancer (NSCLC) (Yageta et al., 2002), and HEK293 cell/neuron co-culture assay (Hoy et al., 2009), and thus, the 4.1N FERM domain is assumed to interact with CAM1. Moreover, as mentioned in the previous subsection, 4.1N is associated with AMPARs through the CTD, a domain that is also 73% identical to the CTD of 4.1B. Given the facts above, interestingly, the 27% amino acid difference in the CTD between 4.1N and 4.1B is enough to switch interaction from AMPAR subunits to NMDAR subunits (Hoy et al., 2009). 4.1N–CAM1 interaction is expressed in the distal uriniferous tubules (not in the proximal uriniferous tubules), whereas 4.1B–CAM4 interaction is detected in the proximal uriniferous tubules (not in the distal uriniferous tubules) (Nagata et al., 2012). Although 4.1N, CAM1, 4.1B, and CAM4 are expressed in many other organs together, such a distinct expression pattern is not observed elsewhere, suggesting that the unique expression pattern is related to unknown roles in each uriniferous tubule (Nagata et al., 2012). Protein 4.1N targets the C-terminus of CAM3 through its FERM domain in neurons, and the association is necessary for CAM3 to recruit protein 4.1N from plasma to the cell to the cell junction on the plasma membrane. The 4.1N–CAM3 interaction is likely to link the F-action cytoskeleton, leading to the regulation of the synaptic architecture and the function in the nervous system (Zhou et al., 2005). 4.1R–CAM1 interaction is recently verified in small-cell lung cancer (SCLC) (Funaki et al., 2021). In SCLC cells NCI-H446, 4.1N, 4.1R, and 4.1G are expressed at the cell–cell contact sites and colocalized with CAM1. In CAM1-knockout NCI-H446 cells, 4.1R expression at the intercellular junctions is lost but localization of 4.1N and 4.1G is not affected, suggesting that 4.1R, but not 4.1N or 4.1G, is recruited to the cell membrane in a CAM1-dependent manner (Funaki et al., 2021).

The 4.1G-deficient nerve demonstrates the 4.1G functions as a transporter for CAM4, leading to morphological and physiological impairments in peripheral nerves (Ohno et al., 2006). 4.1G regulates the cell–cell adhesion between spermatogenic and Sertoli cells by directly interacting with CAM4 in Sertoli cells. 4.1G deficiency alters the extent of localization of CAM4 to the membrane, affecting the intercellular adhesion between Sertoli cells and germ cells and causing male infertility. Defects in every member of the 4.1 family have been shown to underlie function defects or even human disease (Rangel et al., 2017); however, no overt defects other than male infertility are observed in 4.1G-deficient mice (Yang et al., 2011). In addition, the 4.1B −/− null mice develop normally and are fertile (Yi et al., 2005). A possible explanation is that the functional impairment of one 4.1 protein may be compensated by the other 4.1 family members, because these 4.1 proteins are expressed together in many tissues. For example, 4.1N is significantly upregulated in 4.1R-deficient CD4^+^ T cells (Kang et al., 2009). Both 4.1N and 4.1G are upregulated in 4.1R-deficient keratinocytes (Yang et al., 2011). 4.1B-deficient mice have an increase in 4.1R at the axonal paranodes (Einheber et al., 2013). However, an exception has also been reported. The 4.1G–MPP6–CAM4 complex is an adhesion membrane skeleton molecular interaction (Terada et al., 2013). 4.1G and MPP6 colocalize in Schwann cells of the peripheral nervous system (Ohno et al., 2006) and along cell membranes of the spermatogonium and early spermatocytes (Terada et al., 2012) of mice. 4.1G has a specific role in the direct targeting of MPP6 to the Schmidt–Lanterman incisures and the assembly of these subcellular structures (Terada et al., 2012). In 4.1G-knockout mice, an abnormal transport of MPP6 and an altered cell shape are observed in myelinated peripheral nerves; however, specific localization of MPP6 in the seminiferous tubules is unaltered in the 4.1G−/− mice. 4.1B is also found in the seminiferous tubules (Terada et al., 2004) and the intestine (Kamijo et al., 2016) of mice. 4.1B−/− mice do not show a detectable disappearance of the MPP6 targeting in testicular germ cells (Terada et al., 2012) and epithelial cells of the small intestine either. Meanwhile, 4.1N and 4.1G do not compensate for the function of 4.1B in 4.1B−/− epithelial cells (Kamijo et al., 2016). Nonetheless, localization of MPP6 in germ cells is significantly changed in 4.1B/G double-mutant mice compared with that of wild-type mice (Terada et al., 2012). On one hand, further studies are required to solve why such different events happen in different organs and 4.1-gene knockout mice. On the other hand, these findings suggest an ambiguous compensatory mechanism among protein 4.1 family members.

## 4.1N in Cancers

### Non–Small-Cell Lung Cancer

Our previous study shows that 4.1N directly binds to the N region (1–313 residues) of βII spectrin in human bronchial epithelial (HBE) cell lines (Wang et al., 2018). Lateral membrane components 4.1N and βII spectrin are critical for the initial biogenesis and growth/maintenance of the lateral membrane, respectively (Wang et al., 2018). Downregulation of 4.1N leads to a shortened lateral membrane and growth and expansion of the apical membrane. The depleted-4.1N–induced shortened lateral membrane but not the *de novo* biogenesis of the lateral membrane can be restored following the re-expression of 4.1N in HBE cells. Immunofluorescence image analysis suggests that 4.1N is also localized with E-cadherin and β-catenin at the lateral membrane of HBE (Wang et al., 2018).

Previously, we reported a tumor suppressor role of 4.1N linking the PP1/JNK/c-Jun (Wang et al., 2016) and flotillin-1/β-catenin/Wnt (Yang et al., 2016) pathway regulation in NSCLC. Likewise, 4.1B suppresses meningioma growth through regulation of the JNK pathway activation (Robb et al., 2005; Gerber et al., 2006). Depletion of 4.1N leads to neoplastic transformation of HBE cells (Lee et al., 2005; Khatlani et al., 2007; Nitta et al., 2011). 4.1N expression is significantly reduced in NSCLC specimens compared to adjacent normal specimens at both mRNA and protein levels (Zheng et al., 2009; Wang et al., 2016). Endogenous 4.1N expression is negatively correlated with cell metastatic potential and the histological grade of clinical samples in NSCLC. 4.1N suppresses proliferation, migration, adhesion, and invasion of NSCLC cells *ex vivo* and *in vivo* (Wang et al., 2016; Yang et al., 2016). PP1 (Wang et al., 2016) and flotillin-1 ^32^ are 4.1N-interacting molecules. The 4.1N FERM domain mediates the interaction between 4.1N and PP1, while both the FERM and U2 domains mediate between 4.1N and flotillin-1. Protein 4.1 family proteins emerge early in evolution, but the SAB domain is a late evolutionary development and emerges as the invertebrate evolves into a vertebrate. The SAB domain in vertebrates functions to link transmembrane proteins with the spectrin/actin-based cytoskeleton. However, protein 4.1N is a unique 4.1 homology whose SAB domain binds to neither spectrin nor actin (Wang et al., 2013). The U2 domain of amphioxus 4.1 protein is a SAB-like domain and is considered as the primary structure for binding amphioxus protein 4.1 to spectrin and actin (Gimm et al., 2002). We previously reported that both the U2 and FERM domains of 4.1N are implicated in the 4.1N–flotillin-1 interaction for suppressing NSCLC cell proliferation and migration (Yang et al., 2016), uncovering the known binding within the U2 domain. For the other 4.1 homologous protein, 4.1B, the FERM domain functions to target the U2 domain to the cell membrane. Targeting the U2 domain to the plasma membrane is sufficient for 4.1B to suppress meningioma growth (Robb et al., 2005). Nonetheless, the underlying mechanism is unclear.

Tumor suppressor 4.1B is frequently lost in various human cancers, including NSCLC (Wang et al., 2010), breast cancer (Feng et al., 2019), meningiomas (Yu et al., 2002), kidney cancer (Yamada et al., 2006), and prostate cancer (Wong et al., 2007). 4.1B participates in a cascade of NSCLC occurrence and development (Wang et al., 2010; Yu et al., 2015). The expression of 4.1B shows a significant correlation with cancer differentiation and the TNM stage but not with gender, age, and pathological type in NSCLC (Wang et al., 2010). Promoter methylation of the *4.1B* gene predicts poor prognosis in NSCLC (Kikuchi et al., 2005).

### Epithelial Ovarian Cancer

The 4.1N protein expression level was significantly decreased during malignant transformation of epithelial ovarian cancer (EOC). 4.1N protein expression levels are significantly different among type I EOC variants. The loss of 4.1N expression is more related to type II rather than type I EOCs (Xi et al., 2013). 4.1N loss is significantly correlated with a poorer differentiation and aggressive behavior, increased clinical stage progression, lower response to first-line chemotherapeutic treatment, poor overall survival, and progression-free survival in EOC patients (Xi et al., 2013; Wang et al., 2020a). The result from the nude mice model suggests a suppressor role of 4.1N in a unique interesting peritoneal dissemination that is different from lymph node or blood metastases in other cancers (Xi et al., 2013; Wang et al., 2020a). A previous study proposed that 4.1N suppresses EOC’s intraperitoneal dissemination by regulating 4.1N-interacting adhesion molecules and the F-actin cytoskeleton during the epithelial–mesenchymal transition (EMT) (Wang et al., 2020a). Besides, in EOC, a suppressor role of 4.1N in hypoxia-induced EMT and related genes has been demonstrated. The increased expression of hypoxia-induced factor 1α (HIF-1α) accompanied by decreased expression of E-cadherin is a crucial factor of EMT, leading to cancer metastasis and drug resistance (Beavon, 1999; Esteban et al., 2006; Krishnamachary et al., 2006; Moeller et al., 2007; Mak et al., 2010; Rohwer and Cramer, 2011). 4.1N negatively regulates the expression level and nuclear localization of HIF-1α but positively regulates E-cadherin under hypoxic conditions (Zhang et al., 2016; Wang et al., 2020a).

Protein 14-3-3 (14-3-3ζ/δ, 14-3-3γ, and 14-3-3η) is a 4.1N-interacting partner that mediates with the 4.1N FERM domain (key Phe359 residue) (Calinisan et al., 2006). 4.1N negatively regulates 14-3-3 to inhibit EOC aggressiveness *in vitro* and *in vivo* (Wang et al., 2020a). By promoting 14-3-3 degradation and downregulating 14-3-3–dependent Snail expression, 4.1N depletion can decrease the apoptosis of EOC cells (Wang et al., 2020a). The clinical samples’ results indicate that defective expression of 4.1N also results in 14-3-3–dependent epithelial–mesenchymal transition (EMT), anoikis resistance, and entosis. 4.1N, as a single application or combined with 14-3-3 antagonists and entosis inhibitors, is considered as a promising therapeutic approach for treating EOC (Wang et al., 2020a). Besides, the interaction of 4.1N and 14-3-3 is suggested to play an important role in participatory transmembrane protein activities (Na-K ATPase activity, for instance) in the kidney epithelium (Calinisan et al., 2006). Kidney 14-3-3 also interacts with the FERM domain of 4.1B and 4.1R (Calinisan et al., 2006). 4.1B–14-3-3 interaction is involved in the 4.1B-mediated suppression of cell growth in meningioma (Yu et al., 2002); however, disruption of the interaction between 14-3-3 and the 4.1B does not impair the growth-inhibitory effects of 4.1B (Calinisan et al., 2006; Wang et al., 2014).

### Kidney Cancer

Compared with the full-length protein 4.1N, kidney 4.1N lacks small regions of the U2 and SAB domain boundary and most of the U3 region, which may promote or inhibit 4.1N interaction with binding partners (Calinisan et al., 2006). 4.1N has abnormally low expression and is principally associated with cell adhesion in KIRC, resulting in a poor prognosis (Liang et al., 2020). Despite mutation in the FERM domain *via* evaluating 537 sequencing data of KIRC patients, the poor prognosis results from the down-expressed 4.1N rather than the mutation (Liang et al., 2020). 4.1N overexpression in the highly differentiated rat kidney cortical collecting duct clonal cell line leads to cell arrest, whereas deficiency of the kidney 4.1N is supposed to result in proliferative diseases affecting nephrons (Puttini et al., 2005; Calinisan et al., 2006). Cell surface receptor amyloid-beta precursor protein (APP) is also down-expressed in KIRC. Data mining results show that 4.1N and APP synchronically increase cell adhesion, leading to decreased metastasis and invasion in KIRC (Liang et al., 2020). Methylation within the 4.1B gene promoter region is one of the most frequent epigenetic alterations in KIRC and a predictive marker for metastatic recurrence of the surgically resected KIRC (Yamada et al., 2006).

### Breast Cancer

A database mining of 4.1 family members suggests that the 4.1 family can be considered as novel biomarkers and potential therapeutic targets for breast cancer (Feng et al., 2019). 4.1N is a favorable factor for relapse-free survival of breast cancer patients, except for HER2+ subtype patients (Feng et al., 2019). 4.1N and 4.1B are considered as negative regulators of cell adhesion, migration, and invasion in breast cancer. The 4.1N expression level varies in breast cancer cell lines with different metastatic abilities. 4.1N is expressed in low metastatic MCF-7 and middle metastatic T-47D cells, particularly at cell–cell junctions, but not in high metastatic MDA-MB-231 cells. Reintroducing 4.1N into MDA-MB-231 cells results in inhibition of cell adhesion, migration, and invasion (Ji et al., 2012).

Low 4.1B expression is associated with high tumor metastasis in breast cancer (Takahashi et al., 2012). N-methyltransferase 3 is identified as a 4.1B-interacting protein. 4.1B-associated caspase 8–specific activation cooperates with protein methylation to induce apoptosis in breast cancer cells (Jiang and Newsham, 2006). 4.1R and 4.1G mRNA high expressions are correlated with better survival in patients with breast cancer. High expression of 4.1G is significantly associated with longer overall survival in luminal A and protracted relapse-free survival in luminal B subtype breast cancer patients treated with tamoxifen (Feng et al., 2019).

### Prostate Cancer

4.1N mRNA is significantly downregulated in cancerous prostate tissue compared to the benign tissue. Low 4.1N mRNA expression is correlated with earlier biochemical recurrence (Schulz et al., 2010). Methylation is not considered as a cause of downregulated 4.1N mRNA expression in prostate cancer (Schulz et al., 2010). It is hypothesized that the down-expression of 4.1N is correlated with overexpression of oncogenic transcription factor ETS-related gene (ERG) in prostate cancer. The expression of 4.1N mRNA is lower in cancer tissues with elevated ERG mRNA expression than in cancer tissues with close to normal ERG expression (Calinisan et al., 2006); however, the difference is not statistically significant (Schulz et al., 2010). Knockdown of 4.1B increases the metastasis of poorly metastatic cells in an orthotopic model of prostate cancer. 4.1B-deficient mice show increased susceptibility for developing aggressive, spontaneous prostate cancer (Wong et al., 2007).

### Neural Cancer

Protein 4.1s–NuMA interactions are required for NuMA cortical stability and spindle orientation integrity and stretch-induced spindle reorientation (Seldin et al., 2013). 4.1N directly contracts with protein NuMA through the CTD (679–879 residues). 4.1N acts as an antiproliferative mediator of NGF by antagonizing the role of NuMA in mitosis in PC12 cells. When P12 cells are untreated with NGF, most 4.1N pronounces at the periphery of the cell. After NGF treatment, 4.1N translocates to the nucleus to associate with NuMA and prevent the role of NuMA in mitosis. This NGF-induced 4.1N translocation and inhibition of antimitogenic effects can be reversed by overexpressing extranuclear NuMA (Ye et al., 1999). NGF-triggered tyrosine phosphorylation of 4.1N is also identified 10 minutes after the NGF induction. The Trk receptor is supposed to be directly responsible for the 4.1N phosphorylation and the translocation into the nucleus. However, the detailed mechanism is not yet documented (Ye et al., 1999).

The CTD (679–879 residues) of 4.1N is associated with the N terminal (1–23 residues) of phosphoinositide 3 kinase PI3K (PI3K) enhancer PIKE. 4.1N competes with PI3K for binding to PIKE. Endo-nuclear PIKE activates PI3K and induces a G1 cell cycle arrest following NGF treatment in PC12 cells, which can be inhibited by the competitive binding of 4.1N to the PIKE slightly later. A decreased GTPase activity of PIKE is also observed simultaneously (Ye et al., 2000). On the contrary, in the absence of NGF, specific targeting of 4.1N into the nucleus results in G1 phase arrest and an aberrant nuclear morphology in P12 cells (Ye et al., 1999). The reason why 4.1N has opposite roles in regulating G1 phase arrest is unclear. A proteome analysis shows that 4.1N may increase the expression level of the PI3K-associated protein inositol polyphosphate 5-phosphatase (Wang et al., 2020a) that can stimulate cell apoptosis (Kisseleva et al., 2002).

Binding of 4.1R (exons 20 and 21 within the CTD) to NuMA (residues 1788–1810) is observed at the spindle and spindle poles (Mattagajasingh et al., 1999; Huang et al., 2004). The C-terminal 59 residues that constitute the NuMA-interacting domain of 4.1B are highly homologous (93% identity) to that of 4.1R (Parra et al., 2000). cDNA characterization and Western blot analysis show multiple spliceosomes of 4.1B, with functionally relevant heterogeneity in the NuMA-interacting domain (Parra et al., 2000). The interaction between the 4.1G/4.1R-CTD and the NuMA plays a key role in NuMA localization during symmetric (Kiyomitsu and Cheeseman, 2013) and asymmetric (Seldin et al., 2013) cell divisions.

## The Other 4.1N-Related Biological Functions

Protein 4.1N in cardiomyocytes is differentially distributed in subcellular locations. 4.1N is found in the intercalated disc domain of the plasma membrane and intracellular Z-disc cross-traiations but is absent in the lateral face of the plasma member (Pinder et al., 2012). Cardiac 4.1N is speculated to function in crosslink plasma/integral cell membranes with the spectrin–actin cytoskeleton, and cardiac 4.1N deficiency is implicated in links with cardiomyopathies (Taylor-Harris et al., 2005). Patients with deteriorating heart failure undergoing left-ventricular assist device insertion surgery have a higher expression of 4.1N in the Z-disc of the myocardium than patients of stable heart failure (JBirks et al., 2003). Compared with the control group, the heart failure group displayed an increased 4.1R protein expression and decreased levels of protein 4.1N and 4.1G in the myocardial tissue of rats (Ning et al., 2021).

4.1N is considered to be critical for the secretion or transmission of the releasing hormone at the hypothalamic–pituitary gland–reproductive system route. In a 4.1N−/− mice model, 4.1N deficiency shows selective effects on the neuroendocrine and reproductive systems. 4.1N−/− mice are born at a significantly reduced Mendelian ratio and exhibit decreased follicle-stimulating/luteinizing hormone levels, higher mortality, slower growth, and lower weight of reproductive organs in comparison to 4.1N+/+ mice (Wang et al., 2020b).

Furthermore, the 4.1N expression is correlated with expressions of pro-inflammatory cytokines TNF-α, IL1-β, and IL-1 in these patients (JBirks et al., 2003). The underlying mechanisms are not clear. In comparison to Caucasian patients, African-American patients with obesity-related nonalcoholic fatty liver disease have a higher expression of 4.1N gene *EPB41L1* (Stepanova et al., 2010).

## Conclusion

**FIGURE 2:**
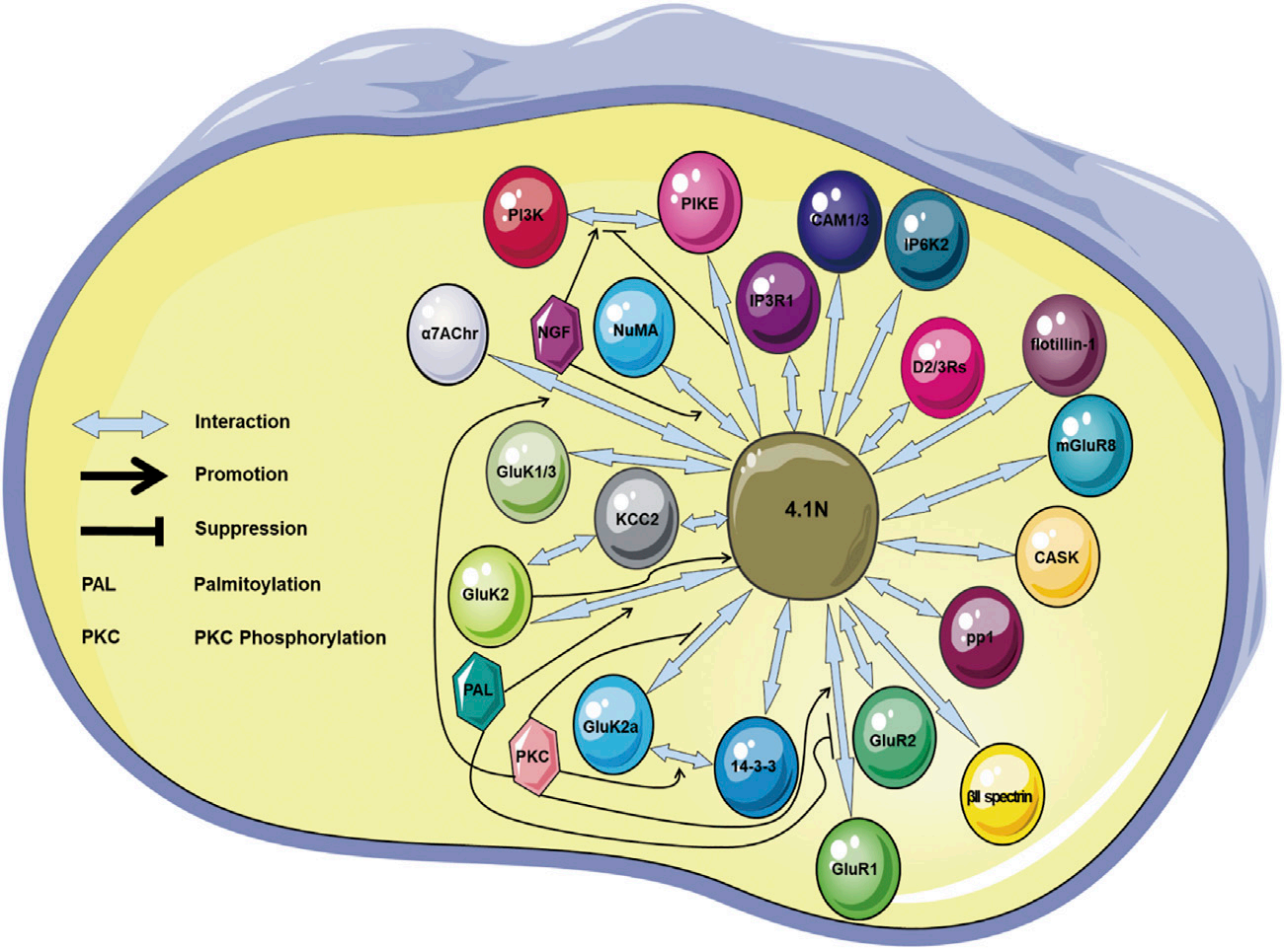
Illustration showing the 4.1N-mediated protein–protein interactions. The proteins IP3R1, GluR1/4, GluK1/2, D2/3Rs, mGluR8, KCC2, CASK, NuMA, PIKE, CADM1/3, βII spectrin, pp1, flotillin-1, 14-3-3, IP6K2, and α7AChr are 4.1N-interacting molecules. The palmitoylation promotes the 4.1N–GluK2 binding but suppresses the binding between 4.1N and GluR1. The phosphorylation promotes the 4.1N–GluK2a, 4.1N–α7AChr, 4.1N–GluR1, and GluK2a–14-3-3 interactions. NGF facilitates the PI3K–PIKE and 4.1N–NuMA interactions, but 4.1N prohibits the PI3K–PIKE interaction. α7AChr, α7 acetylcholine receptor; CAM, cell adhesion molecule; CASK, Ca2+-calmodulin serine kinase; D2/3Rs, D2 and D3 dopamine receptors; IP6K2, inositol hexakisphosphate kinase 2; IP3R1, inositol 1,4,5-trisphosphate receptor type 1; KCC2, K-Cl co-transporter 2; mGluR8, metabotropic glutamate receptor 8; NGF, nerve growth factor; NSCLC, non–small-cell lung cancer; NuMA, nuclear mitotic apparatus; PI3K, phosphoinositide 3 kinase; PIKE, phosphoinositide 3 kinase enhancer.

Scaffolding protein 4.1N that connects multiple components representing critical functions in regulating cell events has been revealed (Table 1; Figure 2). 4.1N localization in various neuronal cells accords with its impact upon synaptic proteins. The 4.1N is essential for trafficking, distribution, stability, and endocytosis of various synaptic proteins, which is important for synaptic plasticity, synaptic transmission, and the spectrin/actin cytoskeleton profile. 4.1N exerts antitumor effects in NSCLC, EOC, breast cancer, prostate cancer, KIRC, and neural cancer. Cells with a lower expression of 4.1N exhibit a higher ability of proliferation and migration. Downregulation or loss of 4.1N has been observed during malignant transformation in some cancers. The 4.1N is correlated with tumor progression, aggressive behaviors in NSCLC and EOC, and chemotherapy resistance in EOC. 4.1N is also related to liver disease (Stepanova et al., 2010), cardiac disease (JBirks et al., 2003; Taylor-Harris et al., 2005; Pinder et al., 2012), nonsyndromic intellectual disability (Hamdan et al., 2011), hereditary Parkinson’s disease (Auburger et al., 2019), and reproductive system disease (Wang et al., 2020b; Wang et al., 2021). The FERM and CTD represent two adaptors where a number of regulations converge on the association of protein 4.1N with its partners, through which 4.1N locates, supports, and coordinates partners in signaling transduction. Moreover, phosphorylation is an important factor or mediator for the association and/or translocation of 4.1N with its contactors. However, much still remains to be elucidated. Particularly, although the 4.1N and other 4.1 homologues share a similar structure and thereby contribute to the mechanochemical properties by jointly binding and regulating the same targets, little is known about the collaboration and delicate balance within a network of 4.1 family members in dynamical modulation of proteins.

**TABLE 1:** 4.1N–protein interactions and functions.

Site of interaction		Function of the interaction	Reference
4.1N (FERM)	GluR1/4 (C-terminus)	Anchoring AMPARs to the actin cytoskeleton, stabilizing AMPAR expression on the excitatory synapse surface, and contributing to synaptic plasticity	Shen et al. (2000), Coleman et al. (2003), Douyard et al. (2007), Lin et al. (2009), White et al., (2016)
4.1N (FERM)	KCC2 (C-terminus)	Regulating neuronal differentiation and migration and promoting dendritic spine development	Li et al. (2007), Horn et al. (2010)
4.1N (FERM)	CAM 3 (C-terminus)	Regulating the function of the cell-to-cell junction and is involved in the morphology and plasticity of the nervous cell	Zhou et al. (2005)
4.1N (FERM)	pp1 (unknown)	Suppressing NSCLC linking the PP1/JNK/c-Jun pathway	Wang et al. (2016)
4.1N (FERM)	14-3-3 (unknown)	Inhibiting EOC aggressiveness by directly binding and accelerating the degradation of 14-3-3	Calinisan et al. (2006), Zhang et al. (2016), Wang et al. (2020a)
4.1N (FERM and U2)	flotillin-1 (unknown)	Suppressing NSCLC linking the flotillin-1/β-catenin/Wnt pathway	Yang et al. (2016)
4.1N (CTD)	IP3R1 (CTT14aa)	Spatiotemporally regulates lateral diffusion of the intracellular Ca^2+^ release channel, mediates neurite formation in neurons, and regulates IP3R1 subcellular localization in MDCK cells	Maximov et al. (2003), Zhang et al. (2003), Fukatsu et al. (2004), Fukatsu et al. (2006), Fiedler and Nathanson (2011)
4.1N (CTD)	α7AChr (316–468 residues)	Increasing *α*7 ACh receptor localization on the membrane surface	Kanno et al. (2013)
4.1N (CTD)	D2/3Rs (N-terminal)	Distributing or stabilizing D2/D3Rs on the cell membrane	Binda et al. (2002), Kabbani and Levenson (2006)
4.1N (CTD)	mGluR8 (C-terminus)	Not determined	Rose et al. (2008)
4.1N (CTD)	NuMA (1440–1913 residues)	Mediating the antiproliferative effect of NGF by antagonizing the role of NuMA in mitosis	Ye et al. (1999), Ilies et al. (2012)
4.1N (CTD)	PIKE (N terminus)	Abolishing the PIKE on the lipid kinase activity of nuclear PI3K	Ye et al. (2000)
4.1N (unknown)	GluK1/2 (C-terminus)	Translocating GluK1/2 receptors to the neuronal plasma membrane and controlling receptor endocytosis	Shanks et al. (2012), Copits and Swanson (2013)
4.1N (unknown)	CAM 1 (unknown)	Not determined	Hoy et al. (2009)
4.1N (unknown)	βII spectrin (N terminus)	Required for sustaining a full function of the lateral membrane in HBE	Wang et al. (2018)
4.1N (unknown)	CASK (C-terminus)	Facilitating F-actin nucleation on neurexins	Cohen et al. (1998), Biederer and Sudhof (2001), Mburu et al. (2006)
4.1N (unknown)	IP6K2 (202–261 residues)	Regulating Purkinje cells and motor coordination	Nagpal et al. (2018)

Recent studies on the 4.1N-mediated interactions and functions in the nerve system and cancer are specifically and comprehensively discussed in this review, thereby providing critical detailed mechanistic insights into the role of 4.1N in disease relationships and also other 4.1 homologues.

## Abbreviations

AMPARs, a-amino-3-hydroxy-5-methyl-4-isoxazole propionate receptors; α7AChr, α7 acetylcholine receptor; CAM, cell adhesion molecule; CASK, Ca2+-calmodulin serine kinase; CTD, C-terminal domain; CTT14aa, C-terminal 14 amino acids of the cytoplasmic tail; D2/3Rs, D2 and D3 dopamine receptors; EOC, epithelial ovarian cancer; FERM, N-terminal 4.1-ezrin-radixin-moesin; HBE, human bronchial epithelial; IP6K2, inositol hexakisphosphate kinase 2; IP3R1, Inositol 1,4,5-trisphosphate receptor type 1; KCC2, K-Cl co-transporter 2; MDCK, Madin–Darby canine kidney; mGluR8, metabotropic glutamate receptor 8; NGF, nerve growth factor; NSCLC, non–small-cell lung cancer; NuMA, nuclear mitotic apparatus; PI3K, phosphoinositide 3 kinase; PIKE, phosphoinositide 3 kinase enhancer; SAB, internal spectrin–actin–binding.
